# Supplementary material for: Stagnation of life expectancy in Korea in 2018: A cause-specific decomposition analysis
Source: PLoS One. 2020 Dec 21;15(12):e0244380. doi: 10.1371/journal.pone.0244380 (PMC7751970; doi:10.1371/journal.pone.0244380)
Supplement: S1 Table — (DOCX) [file pone.0244380.s002.docx]

**S1 Table. The population and number of deaths in Korea between 2003 and 2018**

| Year | Total | | Men | | Women | |
| --- | --- | --- | --- | --- | --- | --- |
|  | Population | Number of deaths | Population | Number of deaths | Population | Number of deaths |
| 2003 | 48,308,386 | 246,441 | 24,235,168 | 135,880 | 24,073,218 | 110,561 |
| 2004 | 48,485,314 | 246,210 | 24,316,613 | 136,233 | 24,168,701 | 109,977 |
| 2005 | 48,683,040 | 245,865 | 24,409,659 | 135,317 | 24,273,381 | 110,548 |
| 2006 | 48,887,027 | 244,154 | 24,506,619 | 134,630 | 24,380,408 | 109,524 |
| 2007 | 49,130,354 | 246,479 | 24,624,127 | 135,663 | 24,506,227 | 110,816 |
| 2008 | 49,404,648 | 246,106 | 24,757,073 | 136,930 | 24,647,575 | 109,176 |
| 2009 | 49,656,756 | 246,894 | 24,876,418 | 137,701 | 24,780,338 | 109,193 |
| 2010 | 49,879,812 | 255,335 | 24,977,164 | 142,314 | 24,902,648 | 113,021 |
| 2011 | 50,111,476 | 257,378 | 25,081,788 | 143,250 | 25,029,688 | 114,128 |
| 2012 | 50,345,325 | 267,172 | 25,187,494 | 147,343 | 25,157,831 | 119,829 |
| 2013 | 50,558,952 | 266,221 | 25,282,928 | 146,589 | 25,276,024 | 119,632 |
| 2014 | 50,763,158 | 267,650 | 25,374,486 | 147,300 | 25,388,672 | 120,350 |
| 2015 | 50,951,719 | 275,854 | 25,458,058 | 150,428 | 25,493,662 | 125,426 |
| 2016 | 51,112,972 | 280,785 | 25,527,815 | 152,520 | 25,585,157 | 128,265 |
| 2017 | 51,230,704 | 285,493 | 25,576,752 | 154,312 | 25,653,952 | 131,181 |
| 2018 | 51,300,880 | 298,777 | 25,601,961 | 161,162 | 25,698,919 | 137,615 |
