# Supplementary material for: Stagnation of life expectancy in Korea in 2018: A cause-specific decomposition analysis
Source: PLoS One. 2020 Dec 21;15(12):e0244380. doi: 10.1371/journal.pone.0244380 (PMC7751970; doi:10.1371/journal.pone.0244380)
Supplement: S2 Table — (DOCX) [file pone.0244380.s003.docx]

**S2 Table. Broad cause-specific contributions to the change in life expectancy in four sub-periods (2003-2006, 2007-2010, 2011-2014, and 2015-2018) in Korea**

| Causes of death | 2003-2006 | | 2007-2010 | | 2011-2014 | | 2015-2018 | |
| --- | --- | --- | --- | --- | --- | --- | --- | --- |
|  | Years | % | Years | % | Years | % | Years | % |
| Infectious diseases (A00-B99) | 0.027 | 1.8 | -0.019 | -1.6 | 0.038 | 3.1 | 0.006 | 0.9 |
| Cancer (C00-D48) | 0.241 | 15.8 | 0.218 | 18.7 | 0.192 | 15.8 | 0.291 | 42.2 |
| Blood & blood-forming organs (D50-D89) | 0.001 | 0.1 | 0.000 | 0.0 | 0.000 | 0.0 | 0.000 | 0.0 |
| Endocrine, nutritional and metabolic diseases (E00-E88) | 0.094 | 6.2 | 0.106 | 9.1 | 0.065 | 5.3 | 0.095 | 13.8 |
| Mental and behavioral disorders (F01-F99) | 0.032 | 2.1 | 0.006 | 0.5 | 0.045 | 3.7 | 0.036 | 5.2 |
| Nervous system (G00-G98) | 0.002 | 0.1 | 0.008 | 0.7 | -0.085 | -7.0 | 0.004 | 0.6 |
| Cardiovascular disease (I00-I99) | 0.506 | 33.2 | 0.448 | 38.4 | 0.349 | 28.6 | 0.218 | 31.6 |
| Respiratory system (J00-J98, U04) | 0.097 | 6.4 | -0.024 | -2.1 | -0.002 | -0.2 | -0.088 | -12.8 |
| Digestive system (K00-K92) | 0.153 | 10.0 | 0.049 | 4.2 | 0.045 | 3.7 | 0.030 | 4.3 |
| Skin and subcutaneous tissue (L00-L98) | -0.001 | -0.1 | 0.000 | 0.0 | 0.004 | 0.3 | -0.001 | -0.1 |
| Musculoskeletal and connective tissue (M00-M99) | 0.027 | 1.8 | 0.014 | 1.2 | 0.021 | 1.7 | 0.009 | 1.3 |
| Genitourinary system (N00-N98) | 0.002 | 0.1 | -0.009 | -0.8 | -0.001 | -0.1 | 0.000 | 0.0 |
| Pregnancy, childbirth and the puerperium (O00-O99) | 0.000 | 0.0 | -0.002 | -0.2 | 0.002 | 0.2 | 0.000 | 0.0 |
| Perinatal deaths (P00-P96) | 0.058 | 3.8 | 0.010 | 0.9 | 0.004 | 0.3 | 0.005 | 0.7 |
| Congenital malformations (Q00-Q99) | 0.036 | 2.4 | 0.016 | 1.4 | -0.001 | -0.1 | 0.008 | 1.2 |
| Ill-defined causes (R00-R99) | -0.001 | -0.1 | 0.325 | 27.8 | 0.275 | 22.6 | -0.016 | -2.3 |
| External causes (V01-Y89) | 0.253 | 16.6 | 0.020 | 1.7 | 0.267 | 21.9 | 0.095 | 13.8 |
| Total life expectancy increase | 1.525 | 100.0 | 1.168 | 100.0 | 1.219 | 100.0 | 0.690 | 100.0 |
