# Supplementary material for: Stagnation of life expectancy in Korea in 2018: A cause-specific decomposition analysis
Source: PLoS One. 2020 Dec 21;15(12):e0244380. doi: 10.1371/journal.pone.0244380 (PMC7751970; doi:10.1371/journal.pone.0244380)
Supplement: S3 Table — (DOCX) [file pone.0244380.s004.docx]

**S3 Table. Detailed cause-specific contributions (for 112 causes of deaths) to the change in life expectancy in 2003-2006, 2007-2010, 2011-2014, and 2015-2018 in Korea**

| Detailed causes of death | 2003-2006 | 2007-2010 | 2011-2014 | 2015-2018 |
| --- | --- | --- | --- | --- |
| Intestinal infectious diseases (A00-A09) | 0.001 | -0.013 | 0.008 | -0.003 |
| Re. certain infectious and parasitic diseases (Re. A00-B99) | -0.003 | -0.002 | 0.005 | 0.000 |
| Tuberculosis (A15-A19) | 0.041 | 0.016 | 0.016 | 0.023 |
| Leptospirosis (A27) | 0.000 | 0.000 | 0.000 | 0.000 |
| Leprosy (A30) | 0.000 | 0.000 | 0.000 | 0.000 |
| Tetanus (A33-A35) | 0.000 | 0.000 | 0.000 | 0.000 |
| Diphtheria (A36) | 0.000 | 0.000 | 0.000 | 0.000 |
| Whooping cough (A37) | 0.000 | 0.000 | 0.000 | 0.000 |
| Scarlet fever (A38) | 0.000 | 0.000 | 0.000 | 0.000 |
| Septicemia (A40-A41) | -0.013 | -0.021 | -0.011 | -0.023 |
| Infections with a predominantly sexual mode of transmission (A50-A64) | 0.000 | 0.000 | 0.000 | 0.000 |
| Typhus fever (A75) | 0.000 | 0.001 | 0.000 | 0.001 |
| Japanese encephalitis (A83.0) | 0.000 | 0.000 | 0.000 | 0.001 |
| Hemorrhagic fever with renal syndrome (A98.5) | 0.000 | 0.001 | 0.000 | 0.001 |
| Measles (B05) | 0.000 | 0.000 | 0.000 | 0.000 |
| Viral hepatitis (B15-B19) | 0.001 | 0.001 | 0.019 | 0.005 |
| Human immunodeficiency virus [HIV] disease (B20-B24) | 0.000 | -0.001 | 0.000 | 0.001 |
| Mumps (B26) | 0.000 | 0.000 | 0.000 | 0.000 |
| Malaria (B50-B54) | 0.000 | 0.000 | 0.000 | 0.000 |
| Malignant neoplasms of lip, oral cavity and pharynx (C00-C14) | 0.004 | 0.005 | -0.001 | 0.007 |
| Malignant neoplasms of digestive organs (C15-C26) | 0.172 | 0.158 | 0.148 | 0.182 |
| Malignant Neoplasms of respiratory and intrathoracic organs (C30-C39) | 0.030 | 0.042 | 0.031 | 0.073 |
| Malignant Neoplasms of bone and articular cartilage (C40-C41) | 0.002 | 0.001 | 0.001 | 0.001 |
| Melanoma and other Malignant Neoplasms of skin (C43-C44) | 0.002 | 0.000 | -0.001 | 0.003 |
| Malignant neoplasms of mesothelial and soft tissue (C45-C49) | 0.000 | -0.003 | 0.001 | 0.001 |
| Malignant neoplasm of breast (C50) | -0.001 | 0.000 | -0.003 | 0.004 |
| Malignant Neoplasms of female genital organs (C51-C58) | 0.012 | 0.006 | 0.004 | 0.005 |
| Malignant Neoplasms of male genital organs (C60-C63) | -0.003 | 0.001 | 0.001 | 0.001 |
| Malignant Neoplasms of urinary tract (C64-C68) | 0.008 | 0.001 | 0.000 | 0.006 |
| M. neoplasms of eye, brain & other parts of central nervous system(C69-C72) | 0.007 | 0.005 | 0.003 | 0.001 |
| Malignant Neoplasm of thyroid gland (C73) | 0.002 | 0.003 | 0.004 | 0.001 |
| Malignant Neoplasms of other endocrine gland (C74-C75) | 0.001 | 0.003 | 0.000 | 0.000 |
| M. neoplasms of ill-defined, secondary & unspecified sites(C76-C80) | 0.009 | 0.007 | 0.006 | 0.001 |
| M. neoplasms of lymphoid, hematopoietic & related tissue (C81-C96) | 0.003 | 0.005 | 0.001 | 0.003 |
| Malignant Neoplasm of independent (primary) multiple sites (C97) | 0.001 | -0.009 | 0.000 | 0.000 |
| Neoplasms of in situ, benign, uncertain or unknown behavior (D00-D48) | -0.008 | -0.008 | -0.002 | 0.002 |
| Re. Diseases of the blood & blood-forming organs (Re. D50- D89) | 0.001 | -0.001 | 0.001 | 0.000 |
| Other anemias (D50-D59, D62-D64) | 0.001 | -0.001 | 0.000 | -0.002 |
| Aplastic anemias (D60,D61) | -0.001 | 0.003 | -0.001 | 0.002 |
| Disorders of thyroid gland (E00-E07) | 0.001 | 0.001 | 0.001 | 0.000 |
| Remainder of endocrine, nutritional & metabolic dis. (Re. E00- E88) | 0.000 | 0.001 | 0.002 | 0.000 |
| Diabetes mellitus (E10-E14) | 0.089 | 0.106 | 0.067 | 0.101 |
| Malnutrition and other nutritional deficiencies (E40-E64) | 0.004 | 0.000 | -0.001 | 0.000 |
| Metabolic disorders (E70-E88) | 0.000 | -0.002 | -0.004 | -0.006 |
| Dementia (F01,F03) | 0.026 | -0.011 | 0.041 | 0.037 |
| Remainder of mental and behavioral disorders (Re. F01- F99) | 0.004 | 0.003 | 0.000 | 0.001 |
| Other organic mental disorders (F04-F09) | -0.001 | 0.002 | 0.001 | 0.000 |
| Mental and behavioral disorders due to use of alcohol (F10) | 0.001 | 0.008 | 0.001 | -0.004 |
| Mental disorder use of psychoactive substances except alcohol(F11-F19) | 0.000 | 0.000 | 0.000 | 0.000 |
| Schizophrenia (F20) | 0.002 | 0.003 | 0.002 | 0.001 |
| Mood [affective] disorders (F30-F39) | 0.000 | 0.001 | 0.000 | 0.000 |
| Inflammatory diseases of the central nervous system (G00-G09) | -0.001 | 0.003 | 0.003 | -0.001 |
| Remainder of diseases of the nervous system (Re. G00- G98) | 0.002 | 0.003 | -0.061 | -0.001 |
| Parkinson's disease (G20-G21) | -0.007 | -0.012 | -0.028 | 0.004 |
| Multiple sclerosis (G35) | 0.000 | 0.000 | 0.000 | 0.000 |
| Epilepsy (G40-G41) | 0.007 | 0.009 | -0.002 | 0.001 |
| Cerebral palsy and other paralytic syndromes (G80-G83) | 0.001 | 0.004 | 0.001 | 0.001 |
| Diseases of the eye and adnexa (H00-H57) | 0.000 | 0.000 | 0.000 | 0.000 |
| Diseases of the ear and mastoid process (H60-H93) | 0.000 | 0.000 | 0.000 | 0.000 |
| Acute rheumatic fever and chronic rheumatic heart diseases (I00-I09) | 0.004 | 0.003 | 0.002 | 0.001 |
| Remainder of diseases of the circulatory system (Re. I00- I99) | 0.004 | -0.001 | 0.000 | 0.000 |
| Hypertensive diseases (I10-I13) | 0.051 | 0.063 | 0.038 | 0.006 |
| Ischemic heart diseases (I20-I25) | -0.019 | 0.140 | 0.063 | 0.074 |
| Pulmonary heart disease & diseases of pulmonary circulation (I26-I28) | -0.001 | -0.004 | 0.000 | -0.001 |
| Other forms of heart diseases (I30-I31, I40,I42, I51) | 0.003 | -0.001 | 0.006 | 0.004 |
| Endocarditis and heart valve disorders (I33-I38) | 0.003 | 0.001 | 0.002 | 0.002 |
| Conduction disorders and cardiac arrhythmias (I44-I49) | -0.002 | -0.040 | -0.014 | -0.004 |
| Heart failure (I50) | 0.001 | -0.019 | 0.053 | -0.020 |
| Cerebrovascular diseases (I60-I69) | 0.463 | 0.301 | 0.199 | 0.154 |
| Atherosclerosis (arteriosclerosis) (I70) | 0.002 | 0.001 | 0.002 | 0.002 |
| Aortic aneurysm and dissection (I71) | -0.003 | 0.005 | -0.003 | -0.001 |
| Remainder of diseases of the respiratory system (Re. J00- J98) | 0.015 | -0.005 | 0.008 | -0.008 |
| Influenza (J09-J11) | 0.001 | -0.004 | -0.001 | -0.010 |
| Pneumonia (J12-J18) | -0.046 | -0.070 | -0.057 | -0.117 |
| Chronic lower respiratory diseases (J40-J47) | 0.132 | 0.071 | 0.042 | 0.058 |
| Pneumoconiosis (J60-J65) | 0.000 | 0.003 | 0.003 | 0.002 |
| Pneumonitis due to solids and liquids (J69) | -0.007 | -0.019 | -0.001 | -0.013 |
| Pulmonary edema (J81) | 0.000 | 0.000 | 0.003 | -0.001 |
| Pyothorax (J86) | 0.000 | 0.000 | 0.001 | 0.000 |
| Remainder of diseases of the digestive system (Re. K00- K92) | -0.004 | -0.012 | 0.006 | -0.001 |
| Diseases of esophagus, stomach and duodenum (K20-K31) | 0.011 | 0.006 | 0.005 | 0.003 |
| Noninfective enteritis and colitis (K50-K52) | 0.000 | 0.002 | 0.000 | 0.000 |
| Paralytic ileus and intestinal obstruction without hernia (K56) | 0.000 | 0.000 | -0.002 | 0.002 |
| Peritonitis (K65) | -0.001 | -0.001 | 0.001 | -0.001 |
| Diseases of liver (K70-K76) | 0.142 | 0.051 | 0.031 | 0.022 |
| Cholelithiasis (K80) | 0.004 | 0.003 | 0.002 | 0.002 |
| Acute pancreatitis and other diseases of pancreas (K85-K86) | 0.001 | 0.000 | 0.002 | 0.002 |
| Diseases of the skin and subcutaneous tissue (L00-L98) | -0.001 | 0.000 | 0.004 | -0.001 |
| Diseases of the musculoskeletal system & connective tissue (M00-M99) | 0.027 | 0.014 | 0.021 | 0.009 |
| Diseases of the genitourinary system (N00-N98) | 0.002 | -0.009 | -0.001 | 0.000 |
| Pregnancy, childbirth and the puerperium (O00-O99) | 0.000 | -0.002 | 0.002 | 0.000 |
| Certain conditions originating in the perinatal period (P00-P96) | 0.058 | 0.010 | 0.004 | 0.005 |
| Congenital malformations and chromosomal abnormalities (Q00-Q99) | 0.036 | 0.016 | -0.001 | 0.008 |
| Re. Symptoms, signs, NEC (Re. R00- R99) | 0.006 | -0.111 | 0.059 | -0.071 |
| Senility (R54) | -0.003 | 0.452 | 0.216 | 0.054 |
| Sudden infant death syndrome (R95) | 0.000 | -0.008 | 0.001 | -0.006 |
| Other sudden death, cause unknown (R96) | -0.004 | -0.007 | -0.001 | 0.006 |
| Transport accidents (V01-V99) | 0.115 | 0.067 | 0.054 | 0.064 |
| Remainder of external causes of morbidity & mortality (Re. V01- Y89) | 0.002 | -0.001 | 0.001 | 0.000 |
| Falls (W00-W19) | 0.027 | 0.051 | 0.005 | 0.000 |
| Exposure to inanimate mechanical forces (W20-W49) | 0.005 | 0.003 | 0.003 | 0.006 |
| Accidental drowning and submersion (W65-W74) | 0.016 | 0.010 | 0.007 | 0.005 |
| Other accidental threats to breathing (W75-W84) | 0.000 | 0.009 | 0.002 | -0.002 |
| Exposure to electric current, radiation & pressure (W85-W99) | 0.001 | 0.002 | 0.002 | -0.001 |
| Exposure to smoke, fire and flames (X00-X09) | 0.019 | 0.002 | -0.001 | 0.000 |
| Exposure to forces of nature (X30-X39) | 0.007 | -0.001 | 0.008 | -0.003 |
| Accidental poisoning by and exposure to noxious substances (X40-X49) | -0.001 | 0.001 | 0.000 | -0.004 |
| Exposure to unspecified factors (X59) | 0.005 | -0.024 | -0.002 | 0.023 |
| Intentional self-harm (X60-X84) | 0.053 | -0.113 | 0.138 | -0.003 |
| Applied (homicide) (X85-Y09) | 0.006 | 0.002 | 0.004 | 0.008 |
| Event of undetermined intent (Y10-Y34) | -0.005 | -0.001 | 0.040 | 0.010 |
| Sequelae of external causes of morbidity and mortality (Y85-Y89) | 0.004 | 0.013 | 0.005 | -0.007 |
