# Supplementary material for: Stagnation of life expectancy in Korea in 2018: A cause-specific decomposition analysis
Source: PLoS One. 2020 Dec 21;15(12):e0244380. doi: 10.1371/journal.pone.0244380 (PMC7751970; doi:10.1371/journal.pone.0244380)
Supplement: Attachment — Submitted filename: notes.pdf [file pone.0244380.s005.pdf]

Review of PONE-D-20-16499

August 11 2020

According to the Human Mortality Database (www.mortality.org), which compounds 50 mostly rich countries and areas, since 1990 a yearly decrease in life expectancy at birth was observed in 17% of cases (16% for males and 21% for females), that is in 208 out of 1257 country-years[1]. Does each of these 208 cases deserve a dedicated study? Certainly not. Does the Korean case stand apart as the only country in this list that has so far never experienced a drop in life expectancy? Probably yes. Should we tread carefully when studying a specific case of a specific year? Assuredly yes. Ignoring this general fact leads the authors to work with blinkers, by focusing on a single year while ignoring the general trends and the effect of variability, eventually having to squeeze every last drop of their results to the point of over-interpreting anecdotal evidences.

The aim of this paper is to investigate the causes of the stagnation of life expectancy in South Korea between 2017 and 2018. It is well conceived and written, the choice of data and methods is relevant, and the analyses are sound. However, the focus of this paper on the specificity of the year 2018 is justified by the fact that this year did not witness any improvement (or barely) in mortality for the first time since modern records. Still, if it can indeed be considered as an unprecedented event, it is part of a general pattern of decreasing rate of improvement in the last 15 years (Figure 1), and the fact that it happened during this particular year has probably as much to do with random fluctuations as with the underlying causes of this trend.

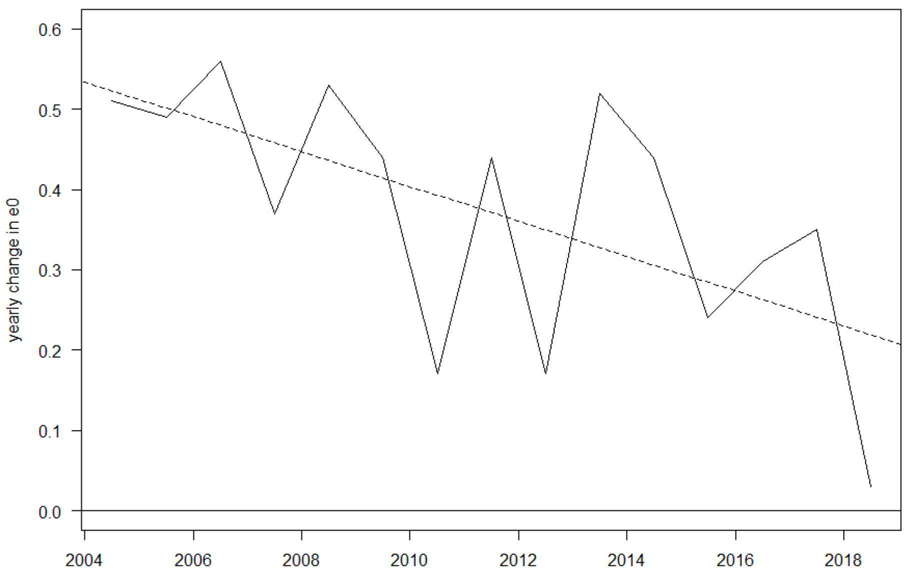

Figure 1: Yearly change in e0 in Korea (total population), HMD 2020

Figure 1 teaches us two things. First, given the overall slowdown in mortality improvements in the last 15 years, if we were to fit a linear trend to the yearly change in e0 (dotted line), we would expect life expectancy to come to a complete halt around the year 2029. Whether or not this really happens at

[1] If we consider instead all years included in the database (the oldest observation being Sweden in 1751), this figure rises to ca. 30% for both sexes. That is to say, despite the clear long-term increase in life expectancy in all of these countries, a decrease was observed in almost every third year.

this particular date, it means that such an eventuality has become more and more probable over time and is bound to happen within the next decade. The authors seem oblivious to this reality and repeatedly state that Korean life expectancy has been steadily increasing to the point of soon becoming the highest in the world (which is not necessarily incompatible with a slowing down of improvements, depending on what happens in other countries like Japan). Considering this trend makes the absence of progress in 2018 much less sudden and unexpected than the authors present it when they write for instance that "The unprecedented increase in life expectancy in Korea suddenly came to a halt in 2018." (line 204).

Secondly, progresses in life expectancy do not come without some natural yearly variability. As shown in Figure 1, the observations are not following that closely the trend line. Between 2003 and 2018, while the mean of annual gains in life expectancy was 0.371, their standard deviation was 0.158, meaning that about half of the yearly change is driven by conjunctural circumstances, i.e. a mix of punctual events like flue epidemics or heat waves, and natural stochasticity (the other half being driven by the causes of the downward trend). For instance, between 2008 and 2013, annual fluctuations in e0 were +0.44, +0.17, +0.44, +0.17, and +0.52, leading to variations (ca. ±0.3) almost as large as the mean annual change across 2003-2018. It is thus misleading to state that "from 2000 to 2017, the rate of increase was consistent" (lines 134-135). Given the level of change in the previous years, it is thus likely that at least part of the drop registered in 2018 was due to conjunctural fluctuations that have little meaning in the general stagnation of life expectancy in Korea.

These important general remarks lead to the following specific necessary improvements in the manuscript.

1. The authors need to put the specific year 2018 in perspective with the general downward (not stable) trend that preceded it. This applies specifically to the introduction, notably by avoiding to present 2018 as an extraordinary year when mortality suddenly stopped improving out of the blue.
2. The authors need to refrain from making comparisons with other cases such as the American and British ones (e.g. lines 66 to 75), which bear no resemblance to the one of Korea (as noted by themselves on lines 198 to 203). It would be more interesting for instance to draw comparisons with other East-Asian countries with high life expectancy.
3. More attention needs to be paid to the dimensions of variability and uncertainty in the data and the results. I suggest for instance considering resampling methods to compute confidence intervals for the decomposition (Bergeron-Boucher et al., 2019), which would give an indication of the importance of stochasticity in the results. Another option could be to divide the period into several sub-periods (e.g. 2000-2005, 2006-2009, 2010-2014 and 2015-2018), which would (1) allow to see the progressive evolution in the mortality patterns rather than opposing the single year 2018 to the mean of all previous years, (2) work with samples of similar size and thus submitted to a similar level of variability, and (3) possibly avoid having to rely on dubious explanations like copycat suicides (Why would this behavior suddenly increase in this particular year? Is there evidence that the suicide of a specific well-known figure was directly followed by a strong increase in the public?), which feels at the moment like an ad-hoc explanation for a conjunctural event.
4. Why not considering harvesting effects in your discussion? The years 2016 and 2017 both registered annual improvements above what was expectable from the general trend (Figure 1). Could the subsequent excess mortality in 2018 be partly due to "overdue" deaths? A similar effect could for instance explain the drop in 2015.

Additionally, I have two requests regarding the data.

1. I would like the authors to be more specific in their appreciation of the data quality, especially for the period 2000-2002. In their documentation, the Human Mortality Database indicate a historical strong underregistration of infant mortality (also mentioned by the authors on lines 97-104). For this reason, the HMD chose to start their series in 2003, not in 2000. Could you explain the reason behind this difference with your study? How do you value the quality of your data between 2000 and 2002, have you explored the trends of mortality before the first birthday, and do you think this could affect your results?
2. What is the proportion of ill-defined causes of death (R00-R99) in the dataset, and how is this proportion evolving over time? Based on another publication, you suggest that strokes might be responsible for a large part of ill-defined deaths (line 314), although this claim is not substantiated in your study, nor the one you cite. Because they are notoriously hard to interpret and their evolution might reflect changes either in etiology or in registration (or coding), ill-defined deaths are typically proportionally redistributed across all other causes of death, unless it is possible to establish a correlation with specific causes of death across time or subnational entities (e.g. Meslé & Vallin, 2012). I suggest that the authors adopt one of these techniques in order to eliminate ill-defined causes of death from the analysis.

Finally, I would like the authors to clarify what they mean in the following two paragaphs of the methods section.

1. I do not understand what they mean by "Life expectancy at birth is calculated by dividing the number of surviving 0-year-olds by the stationary population of 0-year-olds. This study calculated the stationary population of each age based on the surviving number of individuals at each age, the total stationary population beyond a certain age, and the life expectancy at birth for each age category." (lines 111-114). Computing life expectancy does not require working with stationary populations, so I do not understand what was the aim of this approach, nor how it was practically implemented. Was it designed to correct for the under-registration of infant deaths?
2. It is not my understanding that the Arriaga method distinguishes between direct, indirect and interaction effects (lines 119-123). I belive that it only gives one contribution by age group, which can be further decomposed by cause of death using the assumption of proportionality between age-specific death rates and age-specific contributions. I would like the authors to check this point and either use these three effects in their results, or drop this reference that only introduces confusion since only one effect is later presented in the results.

References

Bergeron-Boucher, M.-P., Oeppen, J., Holm, N. V., Nielsen, H. M., Lindahl-Jacobsen, R., & Wensink, M. J. (2019). Understanding differences in cancer survival between populations: A new approach and application to breast cancer survival differentials between Danish regions. *International journal of environmental research and public health, 16*(17), 3093.

Meslé, F., & Vallin, J. (2012). General Trends in Mortality by Cause. In *Mortality and Causes of Death in 20th-Century Ukraine* (pp. 153-172): Springer.
