# Supplementary material for: Stagnation of life expectancy in Korea in 2018: A cause-specific decomposition analysis
Source: PLoS One. 2020 Dec 21;15(12):e0244380. doi: 10.1371/journal.pone.0244380 (PMC7751970; doi:10.1371/journal.pone.0244380)
Supplement: Attachment — Submitted filename: Response to Reviewers.docx [file pone.0244380.s007.docx]

**Authors’ Responses to Comments**

Based on the editor’s and reviewers’ comments, we additionally revised our manuscript. All changes were highlighted in red on the revised manuscript.

**Editorial requests:**

1) there are a very few points in editing to be adjusted - please review that

(OUR RESPONSE) Based on the reviewers’ comment, we revised some expressions in our paper and moved a Table to the appendix with deleting an overlapping Table. Please see our revised version.

2) I agree that data in the paper should be available - Figshare is an option

(OUR RESPONSE) All data used in this study can be accessed via KOSIS ([https://kosis.kr/eng/index/index.do](about:blank)) - Population Statistics Based on Resident Registration, Deaths and Death rates by cause (236 item), sex, and age. However, based on the reviewer’s and editor’s suggestion, we submitted the data for the number of deaths and population released by Statistics Korea.

**Reviewer(s)' Comments to Author:**

**Reviewer: 1**

Please change the 0.0 or 0 in your paper. It is better to read there is a null increase or there no decrease/increase than reading 0.0. Change that.

(OUR RESPONSE) We changed the expression ‘an increase of 0.0 years’ to ‘a null increase’. Please see lines 51 in the revised version with track changes.

You provide no explanation in the text for not writing down that the expectation is already high. Do that. I have a question, I am the reviewer. The reader needs to have all the information and this is a peer review journal. You cannot simply say I don't believe that. It is not the proper way to answer. Provide reasoning for your choices.

(OUR RESPONSE) Based on this comment, we inserted two sentences in the Introduction section (lines 61-64 in the revised version with track changes). “One possible explanation for the stagnation would be that Korea’s life expectancy in 2017 is already high (82.7 years) and has reached its limit. Age- and cause-specific contributions to the life expectancy increase may also provide an answer to such explanations.” We found that the contribution of cardiovascular disease (I00-I99) to life expectancy increase is gradually decreasing over the period 2003-2018, which means that mortality from cerebrovascular disease is reaching its limit. We think that this point would be partly related with the explanation that Korea’s life expectancy is already high. Thus, we also inserted a sentence in the discussion section, “partly supporting an explanation that Korea’s life expectancy is already high” (lines 245-246 in the revised version with track changes).

**Reviewer: 2**

All necessary changes have been made to the manuscript. I am fully satisfied by the modifications applied by the authors, and would like to thank them for their detailed and clear responses.

(OUR RESPONSE) Thank you.

In the new version, some tables and figures are partly overlapping, and it might be a good idea to move some of them (for instance the tables) to the appendix. This is a matter to see with the editorial team though.

(OUR RESPONSE) Since the contents of Table S2 and Table 2 overlapped, Table S2 was deleted and Table 2 was moved to the appendix as Table S2.

For the record, resampling methods are also possible on aggregate data, using a Monte Carlo approach based on a theoretical distribution of the aggregate data (in this case Poisson), but the authors' choice of avoiding that path is understandable given that the division of the observation into several sub-periods was sucessful in clarifying the conclusions.

(OUR RESPONSE) Thank you for this comment. Based on the comment, we modified our study limitation section. Please see lines 273-275 in the revised version with track changes.

Regarding the availability of the data, I let the editor judge, but I assume that providing the age- and cause-specific death rates for the different subperiods would be enough (e.g. as printed tables or a csv file)

(OUR RESPONSE) All data used in this study were provided by KOSIS ([https://kosis.kr/eng/index/index.do](about:blank)) - Population Statistics Based on Resident Registration, Deaths and Death rates by cause (236 item), sex, and age. You can access the data directly from KOSIS, but for your convenience, we submitted the data in xlsx file format.
